# The demographic and treatment options for patients with large cell neuroendocrine carcinoma of the lung

**DOI:** 10.1002/cam4.2188

**Published:** 2019-05-14

**Authors:** Jianjun Gu, Daohui Gong, Yuxiu Wang, Beiyuan Chi, Jun Zhang, Suwei Hu, Lingfeng Min

**Affiliations:** ^1^ Clinical Medical College of Yangzhou University, Department of Respiratory Medicine Subei People’s Hospital Yangzhou China; ^2^ Department of Respiratory Medicine Subei People’s Hospital, Dalian Medical University Yangzhou China; ^3^ The Affiliated Hospital of Yangzhou University, Yangzhou Women and Children Hospital Yangzhou China; ^4^ Nanjing Drum Tower Hospital The Affiliated Hospital of Nanjing University Medicial School Nanjing China

**Keywords:** chemotherapy, large cell neuroendocrine carcinoma, prognosis, radiation, surgery

## Abstract

**Introduction:**

Lung large cell neuroendocrine carcinoma (L‐LCNEC) is a rare, aggressive tumor, for which the optimal treatment strategies for LCNEC have not yet been established. In order to explore how to improve the outcome of prognosis for patients with LCNEC, this study investigated the effect of different treatments based on the data obtained from the Surveillance, Epidemiology, and End Results (SEER) database.

**Methods:**

A total of 2594 LCNEC cases with conditional information were extracted from SEER database. Propensity Score Matching (PSM) method was conducted to reduce possible bias between groups. One‐way ANOVA was used to test the differences of characteristics between groups. Univariate and multivariate Cox proportional hazard models were applied to identify prognostic factors.

**Results:**

Clinicopathologic characteristics including gender, age, TNM stage, T stage, N stage, and M stage were all identified as independent prognostic factors. Surgery benefited stage I, II, and III LCNEC patients’ prognoses. The combination treatment that surgery combining with chemotherapy was the optimal treatment for stage I, II, and III LCENC patients. Compared with palliative treatment, stage IV patients obtained better prognoses with the treatment of radiation, chemotherapy, or chemoradiation. When comparing the effect of the three treatments (radiation, chemotherapy, and chemoradiation) in achieving better prognosis for stage IV patients, chemotherapy alone was better than the other treatments.

**Conclusion:**

Surgery combining with chemotherapy was the optimal treatment for stage I, II, and III LCNEC patients; chemotherapy alone achieves more benefit than the other treatments for stage IV patients.

**Abbreviations**
LCNEC: large cell neuroendocrine carcinoma
PSM: propensity score matching
SEER: Surveillance, Epidemiology, and End Results

## 1 INTRODUCTION

Large cell neuroendocrine carcinoma (LCNEC) of the lung, accounting for 3% of all lung cancer cases, is a rare, aggressive tumor with poor prognosis and high recurrence rate.^1^ LCNEC closely correlated with smoke status, almost 90% of all the cases have smoke history. LCNEC was classified as a subtype of large cell carcinomas according to the World Health Organization (WHO) classification of lung tumors, while, in the 2015 WHO classification, it was classified as a neuroendocrine neoplasm along with small‐cell lung cancer. Considering LCNEC shares many similarities with SCLC, such as therapeutic targets and gene alterations,^2^ SCLC‐based chemotherapy was expected to achieve similar effectiveness in patients with LCNEC. Unfortunately, the reported prognoses of LCNEC treated with SCLC‐based chemotherapy are heterogeneous.^3^, ^4^ By now, no standard treatment regimen has been developed. LCNEC should be treated in a manner similar to that used for small cell lung cancer or similar to NSCLC is still on debating.

Considering the optimum treatment for LCNEC patients remain undefined, to improve prognoses in patients with LCNEC, this study investigated the effect of different treatments for LCNEC based on the data obtained from the SEER database.

## 2 MATERIALS AND METHODS

### 2.1 Database and date extraction items

The SEER database is an opening database containing frequency and survival data. SEER*Stat 8.5.0 software was applied for data extraction. The variables including CS Schema v0204+ (lung), ICD‐0‐3 Hist/behav (8013/3), and AJCC 6th were used to extract the cases diagnosed with LCNEC registered in the SEER database.

The demographic and clinicopathologic characteristics were selected as follows: race, age, gender, grade, AJCC stage, AJCC T stage, AJCC N stage, AJCC M stage, surgery, radiation, chemotherapy, follow‐up time, and outcome status. Based on the information of cases provided by the SEER database, we defined overall survival (OS) as the time from diagnosis to death from any cause, and patients alive were censored at the time of the last recording. We deleted the cases that do not contain all these data and obtained 2594 cases for further analysis.

### 2.2 Propensity score matching (PSM)

A propensity 1:1 matched analysis was conducted to reduce possible bias to a minimum in this study. Propensity scores were calculated using logistic regression model for each patient in the comparing groups. The covariates included in the regression were race, age, gender, grade, AJCC stage, AJCC T stage, AJCC N stage, AJCC M stage, surgery, radiation, and chemotherapy. Patients in two groups were matched based on the propensity score (0.02). Covariates balance between two groups was examined by χ^2^ test. The survival comparisons were then performed for the propensity score‐matched patients using the Kaplan‐Meier method.

### 2.3 Statistical analysis

SPSS (24.0) was used for statistical analysis. Overall survival was estimated using the Kaplan‐Meier method and compared by log‐rank test. One‐way ANOVA was used to test the statistical difference of race, age, gender, grade, AJCC stage, AJCC T stage, AJCC N stage, AJCC M stage, surgery, radiation, and chemotherapy between the groups. Univariate and multivariate Cox proportional hazard models, with hazard ratios (HRs) and 95% confidence intervals (CIs) reported, were applied to identify factors that associated with OS. The values of *P* < 0.05 were considered statistically significant.

## 3 RESULTS

### 3.1 Patients’ characteristics

The characteristics of the 2594 LCNEC patients were shown in Table 1. 2171 LCNEC patients were white people, the elderly patients were accounted for 1848, and there were 1465 males and 1129 females. The patients with stage I, II, III, and IV were 569, 135, 525, and 1365, respectively.

**Table 1:** Patients’ characteristics

Variable	Value (2594)		
	Alive	Dead	Total
Race			
White	463	1708	2171
Black	71	242	313
Others/unknown	24	86	110
Age			
<60	198	548	746
≥60	360	1488	1848
Gender			
Male	284	1181	1465
Female	274	855	1129
Grade			
I	3	9	12
II	10	21	31
III	252	632	884
IV	76	213	289
Unknown	217	1161	1378
TNM			
I	279	290	569
II	46	89	135
III	104	421	525
IV	129	1236	1365
T			
Tx	20	203	223
T0	2	22	24
T1	192	351	543
T2	234	593	827
T3	24	113	137
T4	86	754	840
N			
Nx	7	90	97
N0	344	616	960
N1	54	184	238
N2	118	800	918
N3	35	346	381
M			
M0	429	800	1229
M1	129	1236	1365

### 3.2 Identifying adverse prognosis factors for LCNEC patients

LCNEC is an aggressive tumor with grim prognosis; moreover, the diagnostic rate was increasing in recent years (Figure S1A). It is necessary to explore the factors that influenced long‐term survival of patients with LCNEC. Univariate and multivariate Cox regression analyses were performed to determine prognostic factors (Table 2). The results suggested that race, grade, T1, and N1 were not considered as independent adverse prognostic factors for LCNEC patients. However, other characteristics including gender [male vs female, 1 vs 0.847 (0.775‐0.926)], age [<60 vs ≥ 60, 1 vs 1.396 (1.264‐1.542)], TNM stage{[I vs II, 1 vs 1.525 (1.145‐2.032)]; [I vs III, 1 vs 1.762 (1.444‐2.149)]; [I vs IV, 1 vs 3.831 (3.199‐4.590)]}, T stage {[T0 vs T2, 1 vs 1.638 (1.065‐2.518)]; [T0 vs T3, 1 vs 1.985 (1.250‐3.154)]; [T0 vs T4, 1 vs 2.145 (1.400‐3.285)] ; [T0 vs Tx, 1 vs 1.650 (1.060‐2.569)]}, N stage {[N0 vs N2, 1 vs 1.253 (1.096‐1.432)]; [N0 vs N3, 1 vs 1.433 (1.226‐1.674)]; [N0 vs Nx, 1 vs 1.516 (1.188‐1.934)]}, M stage [M0 vs M1, 1 vs 3.831 (3.199‐4.590)] were all identified as independent prognostic factors.

**Table 2:** Univariate and multivariate analyses for LCNEC patients

Characteristic	Univariate Cox regression		Multivariate Cox regression	
	HR (95% CI)	*P* value	HR (95% CI)	*P* value
Race				
White	1.00 Reference		1.00 Reference	
Black	0.930 (0.813‐1.064)	0.290	0.911 (0.795‐1.044)	0.181
Others	1.013 (0.816‐1.259)	0.904	0.832 (0.669‐1.034)	0.097
Age				
<60	1.00 Reference		1.00 Reference	
≥60	1.306 (1.184‐1.440)	**0.000**	1.396 (1.264‐1.542)	**0.000**
Gender				
Male	1.00 Reference		1.00 Reference	
Female	0.816 (0.747‐0.891)	**0.000**	0.847 (0.775‐0.926)	**0.000**
Grade				
I	1.00 Reference		1.00 Reference	
II	0.760 (0.348‐1.660)	0.492	1.007 (0.460‐2.205)	0.985
III	0.901 (0.467‐1.740)	0.757	1.205 (0.623‐2.332)	0.579
IV	0.974 (0.500‐1.899)	0.938	1.283 (0.657‐2.505)	0.466
Unknown	1.524 (0.791‐2.937)	0.208	1.450(0.751‐2.800)	0.268
TNM				
I	1.00 Reference		1.00 Reference	
II	1.678 (1.323‐2.129)	**0.000**	1.525 (1.145‐2.032)	**0.004**
III	2.591 (2.228‐3.014)	**0.000**	1.762 (1.444‐2.149)	**0.000**
IV	5.488 (4.796‐6.279)	**0.000**	3.831 (3.199‐4.590)	**0.000**
T				
T0	1.00 Reference		1.00 Reference	
T1	0.593 (0.385‐0.913)	**0.017**	1.456 (0.940‐2.256)	0.093
T2	0.831 (0.543‐1.272)	0.395	1.638 (1.065‐2.518)	**0.025**
T3	1.124 (0.712‐1.774)	0.617	1.985 (1.250‐3.154)	**0.004**
T4	1.779 (1.164‐2.720)	**0.008**	2.145 (1.400‐3.285)	**0.000**
Tx	1.640 (1.056‐2.546)	**0.028**	1.650 (1.060‐2.569)	**0.027**
N				
N0	1.00 Reference		1.00 Reference	
N1	1.621 (1.374‐1.913)	**0.000**	1.118 (0.919‐1.359)	0.264
N2	2.357 (2.119‐2.623)	**0.000**	1.253 (1.096‐1.432)	**0.001**
N3	3.076 (2.685‐3.523)	**0.000**	1.433 (1.226‐1.674)	**0.000**
Nx	3.609 (2.884‐4.516)	**0.000**	1.516 (1.188‐1.934)	**0.001**
M				
M0	1.00 Reference		1.00 Reference	
M1	3.373 (3.068‐3.708)	**0.000**	3.831 (3.199‐4.590)	**0.000**

Bold indicates the significance value (*P* < 0.05).

### 3.3 Surgery benefit stage I, II, and III LCNEC patients’ prognosis

**Figure 1:**
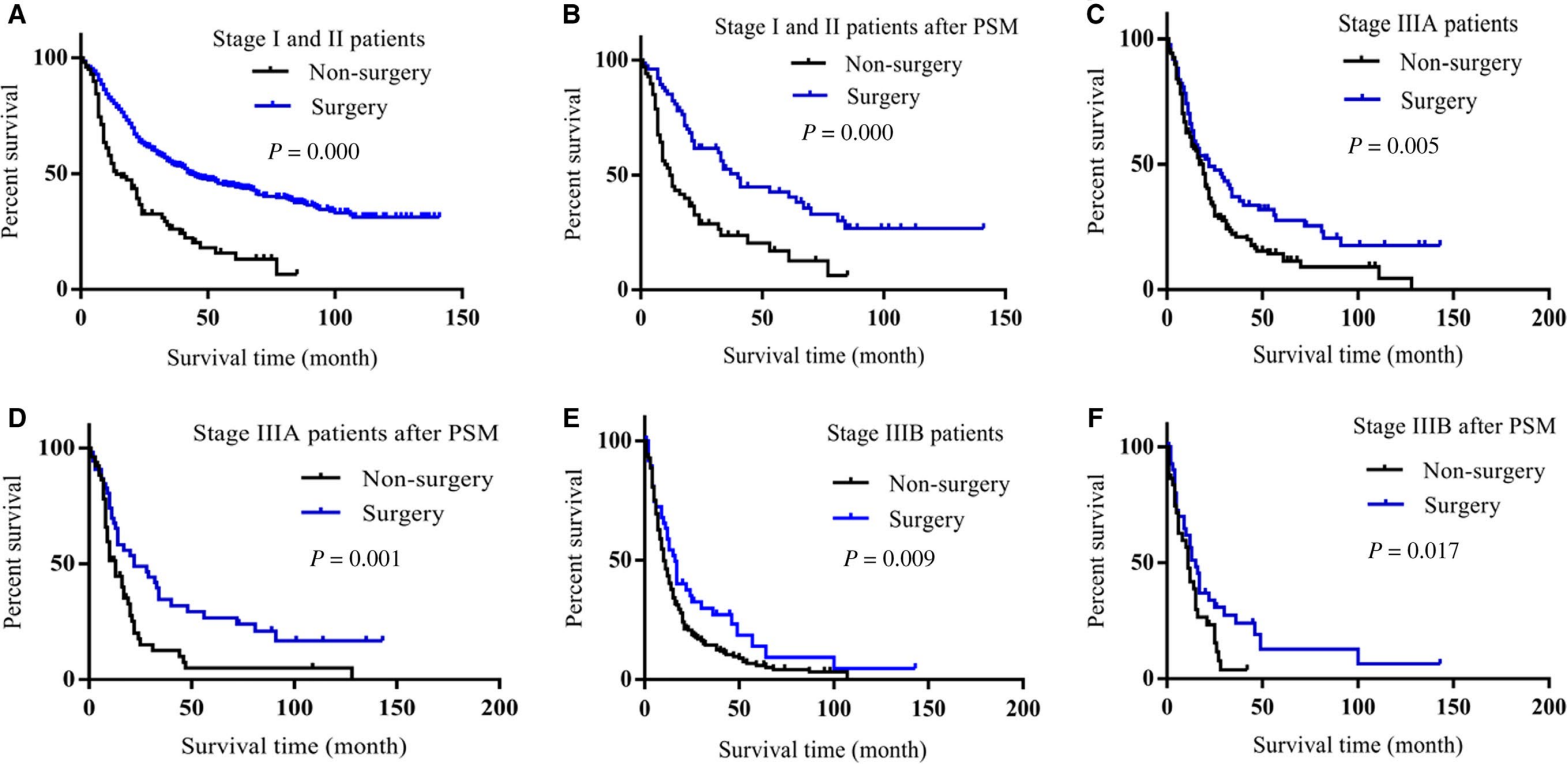
Surgery benefited stage I, II, and III LCNEC patients’ prognoses. A, Surgery patients achieved better prognoses than non‐surgery patients in stage I and II LCNEC patients (*P *= 0.000). B, Surgery patients achieved better prognoses than non‐surgery patients in stage I and II LCNEC patients after PSM was conducted (*P* = 0.000). C, Surgery patients achieved better prognoses than non‐surgery patients in stage III A LCNEC patients (*P* = 0.005). D, Surgery patients achieved better prognoses than non‐surgery patients in stage III A LCNEC patients after PSM was conducted (*P* = 0.001). E, Surgery patients achieved better prognoses than non‐surgery patients in stage III B LCNEC patients (*P* = 0.009). F, Surgery patients achieved better prognoses than non‐surgery patients in stage III B LCNEC patients after PSM was conducted (*P* = 0.017)

When cancer patients are diagnosed at early stage (stage I and II), patients were recommended to perform surgery to obtain better prognosis. To determine whether surgical treatment would benefit the early stage LCNEC patients’ prognoses or not, we firstly divided the stage I and II patients into surgery and non‐surgery group, PSM method was conducted to reduce the differences of variables between groups (Table 3). We found surgery benefit early stage patients’ prognoses (Figure 1A,B). We also found stage III LCNEC patients who undergone surgery had better prognoses than the non‐surgery patients (Figure 1C‐F, Tables S1 and S2). In clinic, the stage IV lung cancer patients are no longer suitable to perform surgery; however, we found that there are still some stage IV LCNEC patients have undergone surgery (Table 4). Because the variable differences such as age (*P* = 0.000), radiation (*P* = 0.029), and chemotherapy (*P* = 0.025) between the groups were exist even PSM method was conducted (Figure S1B,C, Table S3), it is uncertainty that whether surgery would benefit the prognoses or not for stage IV LCNEC patients. The results demonstrated that surgery benefited the stage I, II, and III LCNEC patients; patients at those stages should perform surgery to achieve better prognoses.

**Table 3:** Characteristics among surgical and non‐surgical early stage LCNEC patients before and after propensity score matching

Characteristics	Before PSM analysis		*P*	After PSM analysis		*P*
	Non‐Surgical (n = 107)	Surgical (n = 597)		Non‐Surgical (n = 76)	Surgical (n = 76)	
Race			0.105			0.158
White	85	503		59	49	
Black	20	71		16	21	
Others	2	23		1	6	
Age			**0.001**			0.339
≥60	91	736		61	56	
＜60	16	251		15	20	
Gender			0.946			0.050
Male	55	309		39	27	
Female	52	288		37	49	
Grade			**0.000**			0.181
I	0	3		0	2	
II	1	16		1	3	
III	33	327		30	18	
IV	12	102		11	15	
Unknown	61	149		34	38	
TNM			0.509			0.851
I	84	485		57	58	
II	23	112		19	18	
T			**0.032**			0.628
Tx	0	0		0	0	
T0	0	0		0	0	
T1	44	284		30	32	
T2	51	287		36	36	
T3	12	26		10	8	
T4	0	0		0	0	
N			0.255			0.808
Nx	0	0		0	0	
N0	96	511		67	66	
N1	11	86		9	10	
N2	0	0		0	0	
N3	0	0		0	0	
Radiation			**0.000**			1.000
Yes	62	52		31	31	
No	45	545		45	45	
Chemotherapy			0.366			0.184
Yes	39	191		25	33	
No	68	406		51	43	

Bold indicates the significance value (*P* < 0.05).

**Table 4:** Treatment values of LCNEC patients in different stages

Treatment	Value			
	Stage I	Stage II	Stage III	Stage IV
Palliative treatment	25	8	85	337
Radiation	32	3	33	184
Chemotherapy	6	6	79	346
Chemoradiation	21	6	188	411
Surgery	354	37	47	23
Surgery + Radiation	13	2	5	14
Surgery + Chemotherapy	107	47	41	18
Surgery + Chemoradiation	11	26	47	32

### 3.4 Combination treatment of surgery and chemotherapy benefit stage I, II, III LCNEC patients more than the other treatments

**Figure 2:**
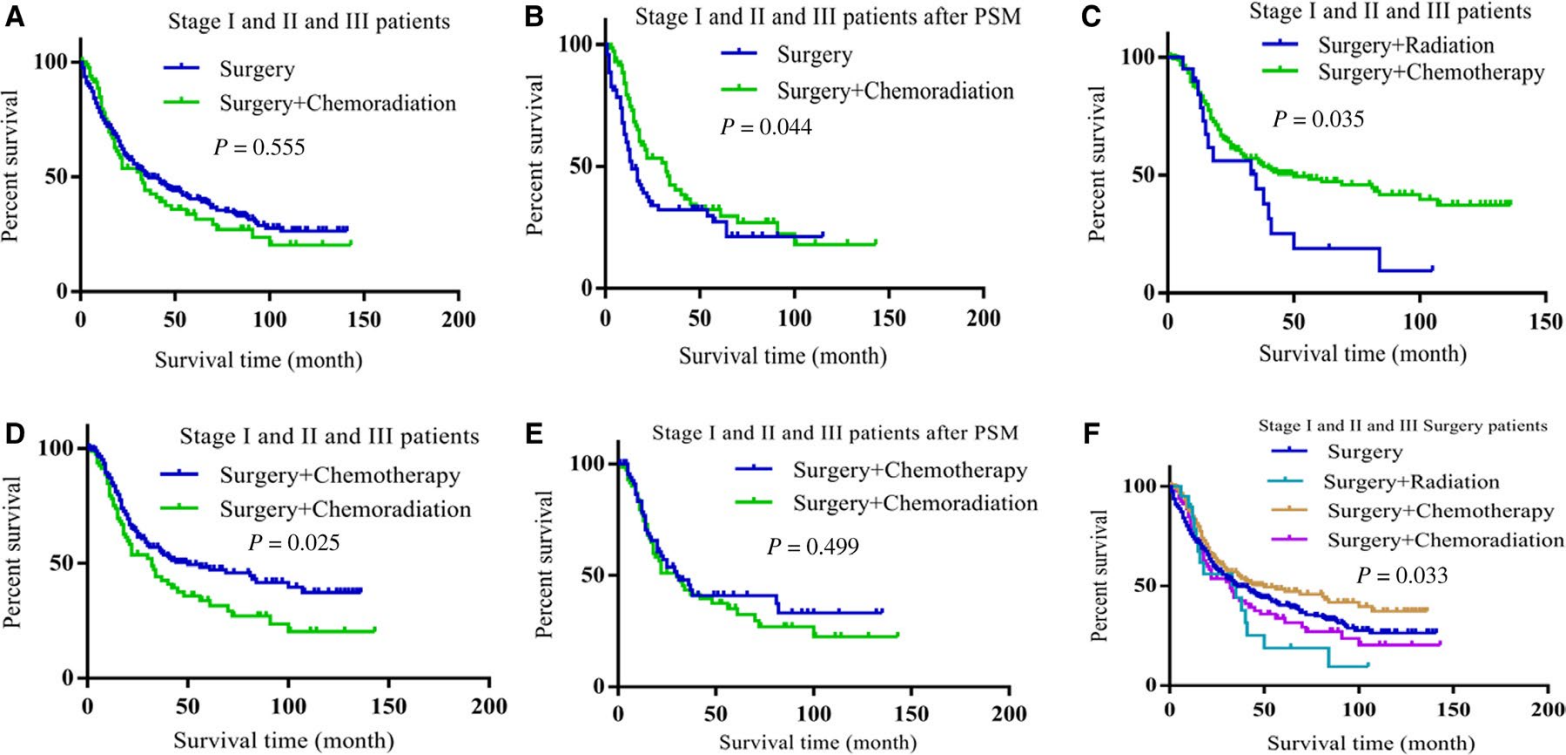
The combination treatment of surgery and chemotherapy benefited stage I, II, and III LCNEC patients better than the other treatments. A, Compared with surgery alone, surgery combining with chemoradiation had no benefit for stage I, II, and III LCNEC patients (*P* = 0.555). B, Surgery combining with chemoradiation achieved better prognosis than surgery alone in stage I, II, and III LCNEC patients after PSM was conducted (*P* = 0.044). C, Surgery combining with chemotherapy achieved better prognosis than surgery combining with radiation (*P* = 0.035). D, Compared with surgery combining with chemoradiation, surgery combining with chemotherapy achieved better prognosis for patients (*P* = 0.025). E, Surgery combining with chemotherapy did not have significant difference when compared with surgery combining with chemoradiation in improving patients’ prognoses after the differences of variables between the groups were reduced (*P* = 0.499). F, Survival comparisons between treatments showed surgery combining with chemotherapy have advantage in improving patients’ prognoses than the other treatments (*P* = 0.033)

LCNEC is an aggressive tumor with high rate of recurrence even after complete surgical resection in its early stage; therefore, surgery alone is not sufficient to treat patients with LCNEC. We firstly compared surgery alone with surgery combining with radiation, surgery combining with chemotherapy and surgery combining with chemoradiation, respectively. When surgery alone compared with the combination treatment of surgery and radiation or the combination treatment of surgery and chemotherapy, there were differences of variables between the groups (Tables S4 and S5); it was uncertainty that whether those combination treatments would achieve better benefit than surgery alone or not (Figure S2A‐D). However, we found, compared with surgery alone, the combination treatment of surgery and chemoradiation achieved better prognoses for stage I, II, and III LCNEC patients (Figure 2A,B, Table 5).

**Table 5:** Characteristics among surgery alone (S) and surgery combining with chemoradiation (S + C + R) in stage I, II, and III LCNEC patients before and after propensity score matching

Characteristics	Before PSM analysis		*P*	After PSM analysis		*P*
	S (n = 438)	S + C + R (n = 84)		S (n = 73)	S + C + R (n = 73)	
Race			0.380			0.891
White	368	73		62	62	
Black	54	7		6	7	
Others	16	4		5	4	
Age			**0.001**			1.000
≥60	338	50		47	47	
＜60	100	34		26	26	
Gender			0.465			0.393
Male	226	47		49	44	
Female	212	37		24	29	
Grade			0.229			0.192
I	3	1		1	1	
II	13	2		2	2	
III	236	51		33	40	
IV	59	13		12	13	
Unknown	127	17		25	17	
TNM			**0.000**			0.411
Stage I	354	11		12	11	
Stage II	37	26		33	21	
Stage III	47	47		28	41	
T			**0.000**			0.263
Tx	0	0		0	0	
T0	2	0		1	0	
T1	232	21		33	20	
T2	172	35		20	28	
T3	11	15		8	14	
T4	21	13		11	11	
N			**0.000**			0.735
Nx	0	0		0	0	
N0	376	27		27	25	
N1	32	20		27	15	
N2	28	36		19	32	
N3	2	1		0	1	

Bold indicates the significance value (*P* < 0.05).

To explore the optimal treatment for stage I, II, and III LCNEC patients, we then compared the prognoses of the three groups (surgery combining with chemotherapy, surgery combining with radiation, and surgery combining with chemoradiation), respectively. We found, compared with the combination treatment of surgery and radiation, surgery combining with chemotherapy showed advantage to improve patients’ prognoses (Figure 2C, Table 6); however, addition of radiation did not achieve better prognosis (Figur2D‐F, Table 7). The results demonstrated that the optimal treatment for stage I, II, and III LCNEC patients was surgery combining with chemotherapy.

**Table 6:** Characteristics among surgery combining with radiation (S + R) and surgery combining with chemotherapy (S + C) in stage I, II, and III LCNEC patients

Characteristics	Before PSM analysis		*P*
	S + R (n = 20)	S + C (n = 195)	
Race			0.716
White	16	167	
Black	2	20	
Others	2	8	
Age			0.164
≥60	15	115	
＜60	5	80	
Gender			0.061
Male	7	111	
Female	13	84	
Grade			0.199
I	0	0	
II	0	4	
III	9	108	
IV	5	45	
Unknown	6	38	
TNM			0.260
Stage I	13	107	
Stage II	2	47	
Stage III	5	41	
T			0.833
Tx	0	1	
T0	0	0	
T1	7	52	
T2	7	115	
T3	5	13	
T4	1	14	
N			0.338
Nx	0	0	
N0	15	112	
N1	4	45	
N2	1	27	
N3	0	1	

**Table 7:** Characteristics among surgery combining with chemotherapy (S + C) and surgery combining with chemoradiation (S + C + R) in stage I, II, and III LCNEC patients before and after propensity score matching

Characteristics	Before PSM analysis		*p*	After PSM analysis		*P*
	S + C (n = 195)	S + C + R (n = 84)		S + C (n = 73)	S + C + R (n = 73)	
Race			0.691			0.633
White	167	73		66	64	
Black	20	7		4	5	
Others	8	4		3	4	
Age			0.932			0.407
≥60	115	50		39	44	
＜60	80	34		34	29	
Gender			0.881			0.511
Male	111	47		35	39	
Female	84	37		38	34	
Grade			0.795			0.488
I	0	1		0	1	
II	4	2		2	2	
III	108	51		48	46	
IV	45	13		12	10	
Unknown	38	17		11	14	
TNM			**0.000**			0.287
Stage I	107	11		11	11	
Stage II	47	26		37	24	
Stage III	41	47		25	38	
T			**0.015**			**0.000**
Tx	1	0		0	0	
T0	0	0		0	0	
T1	52	21		32	14	
T2	115	35		29	33	
T3	13	15		11	14	
T4	14	13		1	12	
N			**0.000**			0.065
Nx	0	0		0	0	
N0	122	27		18	25	
N1	45	20		34	19	
N2	27	36		21	28	
N3	1	1		0	1	

Bold indicates the significance value (*P* < 0.05).

**Figure 3:**
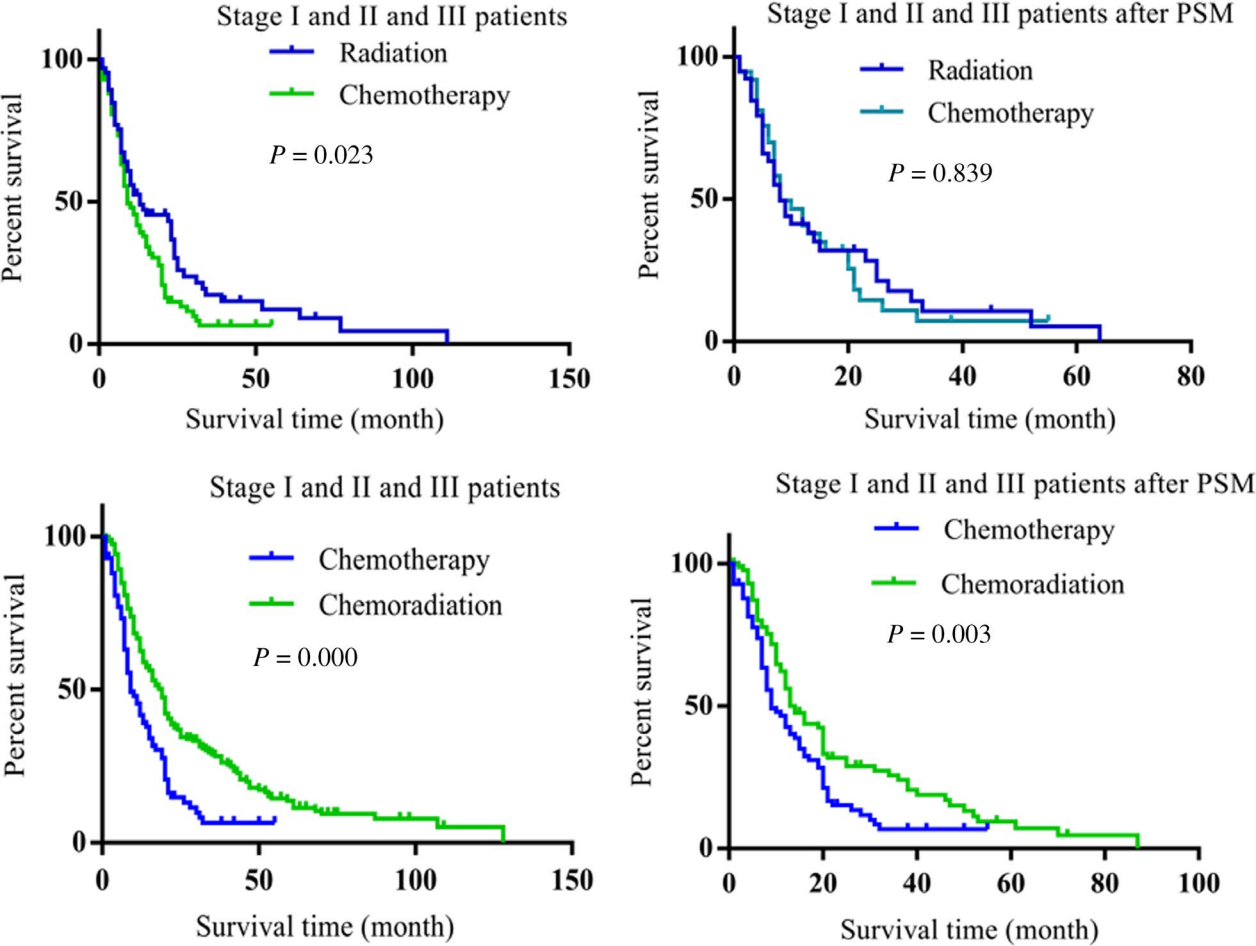
The effect of treatments in non‐surgical stage I, II, and III LCNEC patients. A, Radiation achieved better benefit than chemotherapy for the non‐surgical stage I, II, and III LCNEC patients (*P* = 0.023). B, After the differences of variables between the groups were reduced, compared with chemotherapy, radiation did not showed advantage in proving patients’ prognoses (*P* = 0.839). C, Chemoradiation achieved better prognosis than chemotherapy alone (*P* = 0.000). D, Chemoradiation showed advantage than chemotherapy in improving patients’ prognoses after PSM method was conducted (*P* = 0.003)

Although surgery benefit stage I, II, and III LCNEC patients’ prognoses, there were still some patients did not perform surgery (Table 4). To achieve better prognosis for non‐surgery stage I, II, and III patients, we compared the effect of palliative treatment, radiation, chemotherapy, and chemoradiation for those patients; the prognoses of the under treated patients were better than the palliative treatment group (Figure S3A‐F, Tables S6‐S8). There was no difference between chemotherapy and radiation in proving patients’ prognoses (Figure 3A,B, Table 8). Combination treatment of radiation and chemotherapy achieved better prognosis than chemotherapy alone (Figure 3C,D, Table 9). Interestingly, when compared the combination treatment of radiation and chemotherapy with radiation alone, the combination treatment did not show advantage to achieve better prognoses for patients (Figure S4A,B, Table S9).

**Table 8:** Characteristics among chemotherapy (C) and radiation (R) in stage I, II, and III LCNEC patients before and after propensity score matching

Characteristics	Before PSM analysis		*P*	After PSM analysis		*P*
	R (n = 68)	C (n = 91)		R (n = 41)	C (n = 41)	
Race			0.751			0.883
White	54	70		33	33	
Black	11	16		6	7	
Others	3	5		2	1	
Age			0.224			0.538
≥60	55	66		32	34	
＜60	13	25		9	7	
Gender			0.280			0.513
Male	36	56		23	20	
Female	32	35		18	21	
Grade			0.277			0.243
I	1	0		1	0	
II	1	1		0	0	
III	21	32		13	16	
IV	6	15		4	7	
Unknown	39	43		23	18	
TNM			**0.000**			0.888
Stage I	32	6		6	6	
Stage II	3	6		3	2	
Stage III	33	79		32	33	
T			**0.000**			0.610
Tx	6	7		6	1	
T0	1	0		1	0	
T1	21	6		7	3	
T2	21	23		8	14	
T3	3	8		3	2	
T4	16	47		16	21	
N			**0.000**			0.737
Nx	1	2		1	2	
N0	36	17		10	10	
N1	2	4		2	3	
N2	21	43		20	15	
N3	8	25		8	11	

Bold indicates the significance value (*P* < 0.05).

**Table 9:** Characteristics among chemotherapy (C) and chemoradiation (C + R) in stage I, II, and III LCNEC patients before and after propensity score matching

Characteristics	Before PSM analysis		*P*	After PSM analysis		*P*
	C (n = 91)	C + R (n = 215)		C (n = 89)	C + R (n = 89)	
Race			0.503			0.846
White	70	174		69	68	
Black	16	33		15	16	
Others	5	8		5	5	
Age			0.521			0.203
≥60	66	148		64	56	
＜60	25	67		25	33	
Gender			0.223			0.650
Male	56	116		54	51	
Female	35	99		35	38	
Grade			0.178			0.754
I	0	1		0	1	
II	1	1		1	1	
III	32	68		30	35	
IV	15	22		15	10	
Unknown	43	123		43	42	
TNM			0.185			0.675
Stage I	6	21		6	4	
Stage II	6	6		6	5	
Stage III	79	188		77	80	
T			**0.019**			0.947
Tx	7	12		7	7	
T0	0	2		0	0	
T1	6	38		6	6	
T2	23	59		23	21	
T3	8	17		8	13	
T4	47	87		45	42	
N			0.566			1.000
Nx	2	2		2	0	
N0	17	42		15	20	
N1	4	8		4	7	
N2	43	124		43	45	
N3	25	39		25	17	

Bold indicates the significance value (*P* < 0.05).

### 3.5 Chemotherapy alone benefited stage IV LCNEC patients more than the other treatments

**Figure 4:**
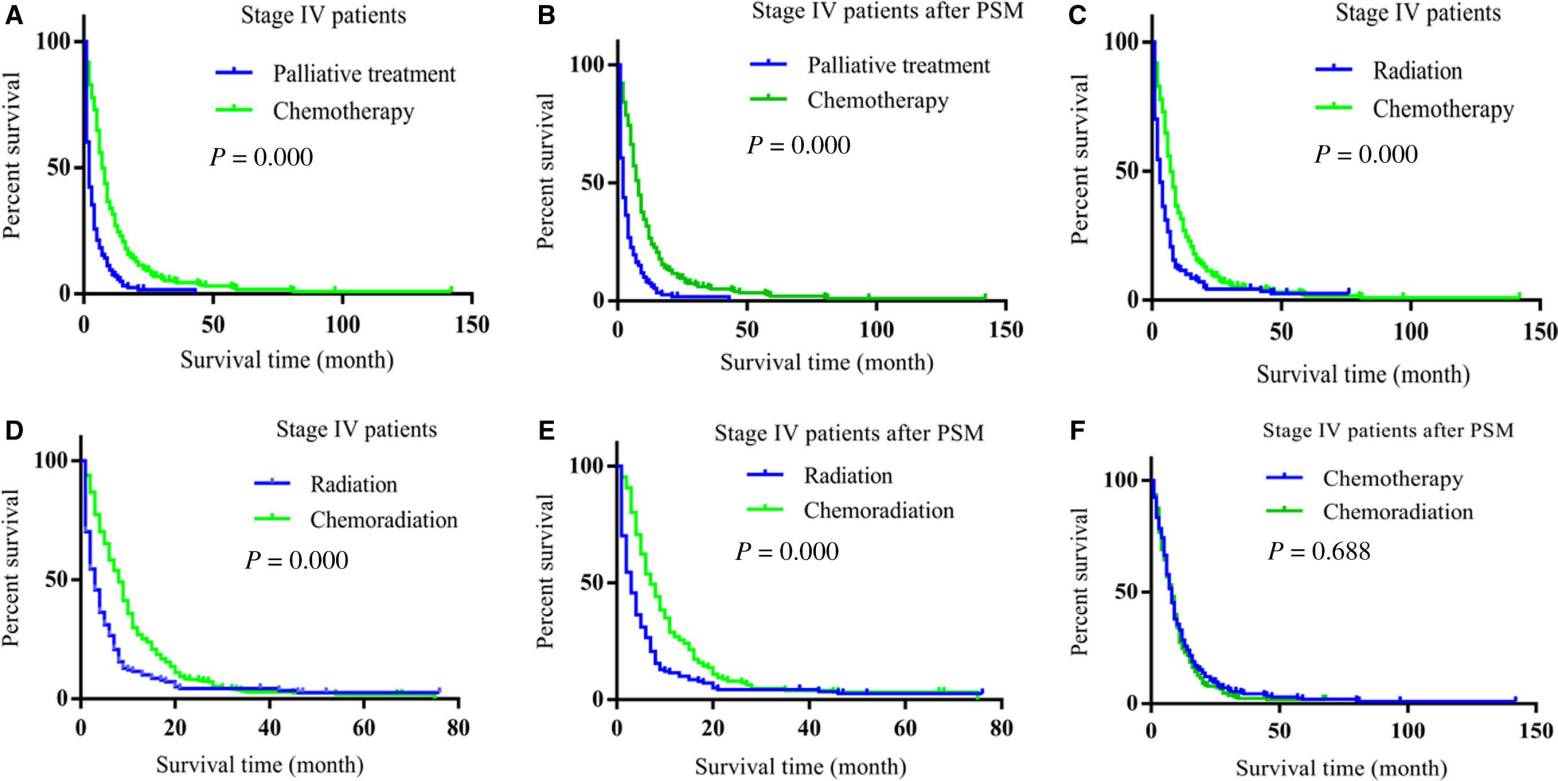
Chemotherapy alone achieved better prognosis than the other treatments in Stage IV LCNEC patients. A, Chemotherapy achieved better prognosis than palliative treatment (*P* = 0.000). B, Chemotherapy achieved better prognosis than palliative treatment after PSM was conducted (*P* = 0.000). C, Chemotherapy achieved better prognosis than radiation treatment (*P* = 0.000). D, Chemoradiation achieved better prognosis than radiation (*P* = 0.000). E, Chemoradiation achieved better prognosis than radiation after PSM was conducted (*P* = 0.000). F, Chemoradiation did not have advantage than chemotherapy alone in proving patients’ prognoses (*P* = 0.688)

As shown in Table 4, the main treatments for stage IV patients were palliative treatment, chemotherapy, radiation, and chemoradiation, we attempted to explore the better treatment for the late stage patients. Compared with palliative treatment, chemotherapy achieved better OS (Figure 4A). To reduce the difference of variable between the groups (age, *P* = 0.030), PSM method was conducted, 308 patients were matched. After PSM, variables between the two groups had no significant differences (Table 10). Chemotherapy treatment has longer OS than palliative treatment (Figure 4B). Furthermore, radiation (Figure S4C, Table S10) and chemoradiation (Figure S4D, Table S11) also achieved better prognoses than palliative treatment.

**Table 10:** Characteristics among palliative treatment (P) and chemotherapy (C) in stage IV LCNEC patients before and after propensity score matching

Characteristics	Before PSM analysis		*P*	After PSM analysis		*P*
	P (n = 337)	C (n = 411)		P (n = 308)	C (n = 308)	
Race			0.536			0.789
White	294	292		266	267	
Black	31	35		30	27	
Others	12	16		12	14	
Age			**0.030**			0.843
≥60	271	254		243	245	
＜60	66	92		65	63	
Gender			0.842			0.934
Male	203	211		194	193	
Female	134	135		114	115	
Grade			0.067			0.604
I	0	4		0	4	
II	1	2		1	0	
III	67	87		66	74	
IV	23	30		21	23	
Unknown	247	223		220	207	
T			0.970			1.000
Tx	54	60		50	50	
T0	4	3		4	3	
T1	34	29		30	24	
T2	80	93		76	84	
T3	13	13		12	12	
T4	152	148		136	135	
N			0.654			1.000
Nx	35	21		31	18	
N0	58	54		49	67	
N1	31	24		30	21	
N2	149	157		139	140	
N3	64	90		59	82	

Bold indicates the significance value (*P* < 0.05).

To determine which one of the treatments (chemotherapy, radiation, chemoradiation) benefits more for the late stage patients, we firstly compared radiation with chemotherapy. Chemotherapy benefited patients more than radiation (Figure 4C, Table 11). Then, we compared radiation with chemoradiation after PSM, 184 patients were matched (Table S12). As shown in Figure 4D,E, chemoradiation obtained better benefit than radiation alone. While compared with chemotherapy alone, the combination treatment chemoradiation did not achieve more benefit (Figure 4F, Figure S4E, Table S13). The results demonstrated that chemotherapy alone was the better treatment than palliative treatment, radiation, and chemoradiation for the stage IV LCNEC patients.

**Table 11:** Characteristics among radiation (R) and chemotherapy (C) in stage IV LCNEC patients before propensity score matching

Characteristics	Before PSM analysis		*P*
	R (n = 184)	C (n = 346)	
Race			0.301
White	151	292	
Black	24	35	
Others	9	16	
Age			0.503
≥60	140	254	
＜60	44	92	
Gender			0.111
Male	99	211	
Female	85	135	
Grade			0.565
I	0	4	
II	1	2	
III	42	87	
IV	14	30	
Unknown	127	223	
T			0.384
Tx	23	60	
T0	3	3	
T1	19	29	
T2	49	93	
T3	10	13	
T4	80	148	
N			0.066
Nx	13	21	
N0	47	54	
N1	16	24	
N2	77	157	
N3	31	90	

## 4 DISCUSSION

The optimal treatment strategies for LCNEC patients have not yet been established. In order to improve prognoses in patients with LCNEC, this study investigated the effect of different treatments based on the data obtained from the SEER database. We found that age, gender, TNM stage, T stage, N stage, and M stage were all independent prognostic factors. Surgery benefited stage I, II, and III LCNEC patients’ prognoses. Surgery combining with chemotherapy was the optimal treatment for stage I, II, and III LCNEC patients. Chemotherapy alone achieved better prognosis than palliative treatment, radiation, or chemoradiation for stage IV LCNEC patients.

Surgical treatment can achieve satisfactory results for suitable patients. As for LCNEC, the patients who suit to perform surgery have no standard by now. Surgical resection was indicated for stage I and II patients to obtain better prognosis.^5^ However, the 1‐year OS rate of stage I, II, and III ALCNEC patients who underwent surgery was better (88.9%) than those who did not undergo surgery (51.9%).^6^ Except the stages reported before, in this study, we also found stage III B LCNEC patients achieved benefit upon surgical treatment. Comparing with previous studies, tumor patients exhibiting both LCNEC and the other kind of tumors as well as the lung metastasis tumors were removed; all the patients analyzed in this study were pure LCNEC patients. Moreover, a bigger cohort of patients was analyzed, and the differences of variables between the groups that may influence the effect of surgery for patients’ prognoses were reduced. Thus, we demonstrate that stage I, II, and III LCNEC patients should perform surgery to achieve better prognosis.

LCNEC is an aggressive tumor with high rate of recurrence even after complete surgical resection in its early stage;^7^ therefore, surgery alone is not sufficient to treat patients with LCNEC, and adjuvant treatment such as chemotherapy or radiation is necessary. Prophylactic cranial irradiation could decrease the incidence of brain metastasis and improve survival rate in patients with SCLC.^8^ Pulmonary neuroendocrine carcinoma patients with brain metastasis could be effectively treated with either whole‐brain radiation therapy or stereotactic radiosurgery (SRS).^9^ However, radiation did not make any benefit in improving LCNEC patients’ prognosis.^10^ Chemoradiation achieved better overall response rate than chemotherapy alone;^11^ unlike the result found in literature, in our study, we found that chemoradiation did not make may benefit in proving stage I, II, and III surgery patients’ prognoses or stage IV patients’ prognoses. The effect of radiation for LCNEC patients is limited and should be reconsidered thoroughly. Contrast with radiation, chemotherapy showed significant advantage. For example, when patients were diagnosed at stage I, II, and III, surgery combining with chemotherapy was the optimal treatment; in stage IV patients, chemotherapy alone achieved better prognosis than the others treatment. Our study demonstrated advantageous position of chemotherapy in improving patients’ prognoses for LCNEC.

In conclusion, through this study, we recommend that stage I, II, and III LCNEC patients should perform surgery to obtain better prognoses, surgery combining with chemotherapy is the optimal treatment for stage I, II, and III LCNEC patients, and chemotherapy alone is better than the other treatments for stage IV patients.

## CONFLICT OF INTEREST

There is no conflict of interest in this manuscript.

## Data Availability

The datasets used during the current study are available from the corresponding author on reasonable request.

## References

[1] Eichhorn F , Dienemann H , Muley T , Warth A , Hoffmann H . Predictors of survival after operation among patients with large cell neuroendocrine carcinoma of the lung. Ann Thorac Surg. 2015;99:983‐989. 10.1016/j.athoracsur.2014.10.015. 25596870

[2] Miyoshi T , Umemura S , Matsumura Y , et al. Genomic profiling of large‐cell neuroendocrine carcinoma of the lung. Clin Cancer Res. 2017;23:757‐765. 10.1158/1078-0432.CCR-16-0355. 27507618

[3] Naidoo J , Santos‐Zabala ML , Iyriboz T , et al. Large cell neuroendocrine carcinoma of the lung: clinico‐pathologic features, treatment, and outcomes. Clin Lung Cancer. 2016;17:e121‐e129. 10.1016/j.cllc.2016.01.003. 26898325 PMC5474315

[4] Sun J‐M , Ahn M‐J , Ahn JS , et al. Chemotherapy for pulmonary large cell neuroendocrine carcinoma: similar to that for small cell lung cancer or non‐small cell lung cancer? Lung Cancer. 2012;77:365‐370. 10.1016/j.lungcan.2012.04.009. 22579297

[5] Kawase A , Nagai K . Treatment strategy for neuroendocrine carcinoma of the lung. Gan To Kagaku Ryoho. 2009;36:1619‐1622. 19838019

[6] Ustaalioglu B , Ulas A , Esbah O , et al. Large cell neuroendocrine carcinoma: retrospective analysis of 24 cases from four oncology centers in Turkey. Thorac Cancer. 2013;4:161‐166. 10.1111/j.1759-7714.2012.00129.x. 28920204

[7] Matsuura N , Nakashima N , Igai H , et al. Prognosis of surgically treated large cell neuroendocrine carcinoma. KyobuGeka. 2011;64:187‐190. 21404553

[8] Cao KJ , Huang HY , Tu MC , et al. Long‐term results of prophylactic cranial irradiation for limited‐stage small‐cell lung cancer in complete remission. Chin Med J (Engl). 2005;118:1258‐1262. 16117878

[9] Kotecha R , Zimmerman A , Murphy ES , et al. Management of brain metastasis in patients with pulmonary neuroendocrine carcinomas. Technol Cancer Res Treat. 2016;15:566‐572. 10.1177/1533034615589033. 26041398

[10] Rieber J , Schmitt J , Warth A , et al. Outcome and prognostic factors of multimodal therapy for pulmonary large‐cell neuroendocrine carcinomas. Eur J Med Res. 2015;20:64 10.1186/s40001-015-0158-9. 26272455 PMC4536693

[11] Shimada Y , Niho S , Ishii G , et al. Clinical features of unresectable high‐grade lung neuroendocrine carcinoma diagnosed using biopsy specimens. Lung Cancer. 2012;75:368‐373. 10.1016/j.lungcan.2011.08.012. 21920624

