# Supplementary figures and images for: The demographic and treatment options for patients with large cell neuroendocrine carcinoma of the lung
Source: Cancer Med. 2019 May 14;8(6):2979–93. doi: 10.1002/cam4.2188 (PMC6558599; doi:10.1002/cam4.2188)

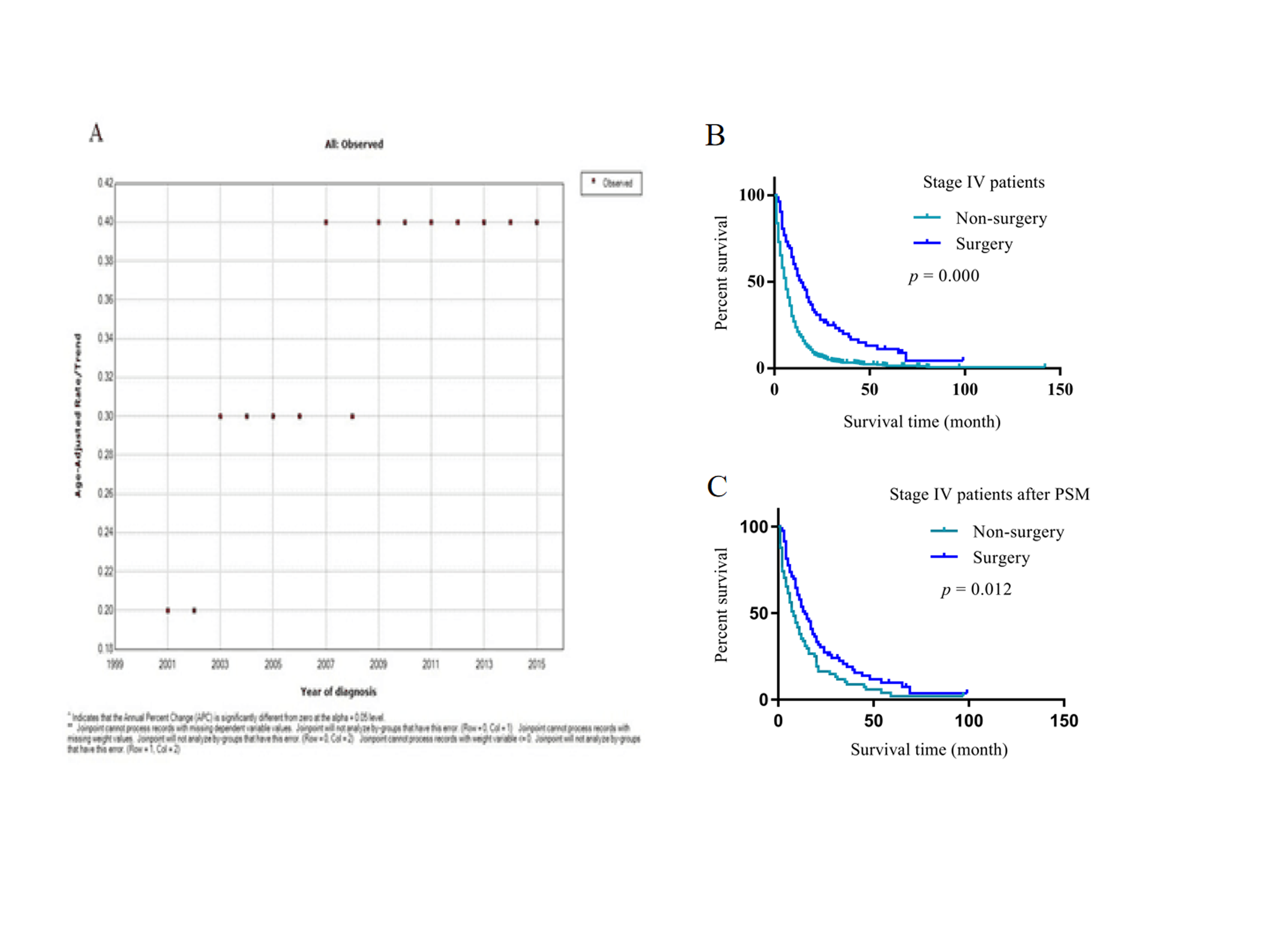

Supplement: Supplementary file 1 [file CAM4-8-2979-s001.tif]

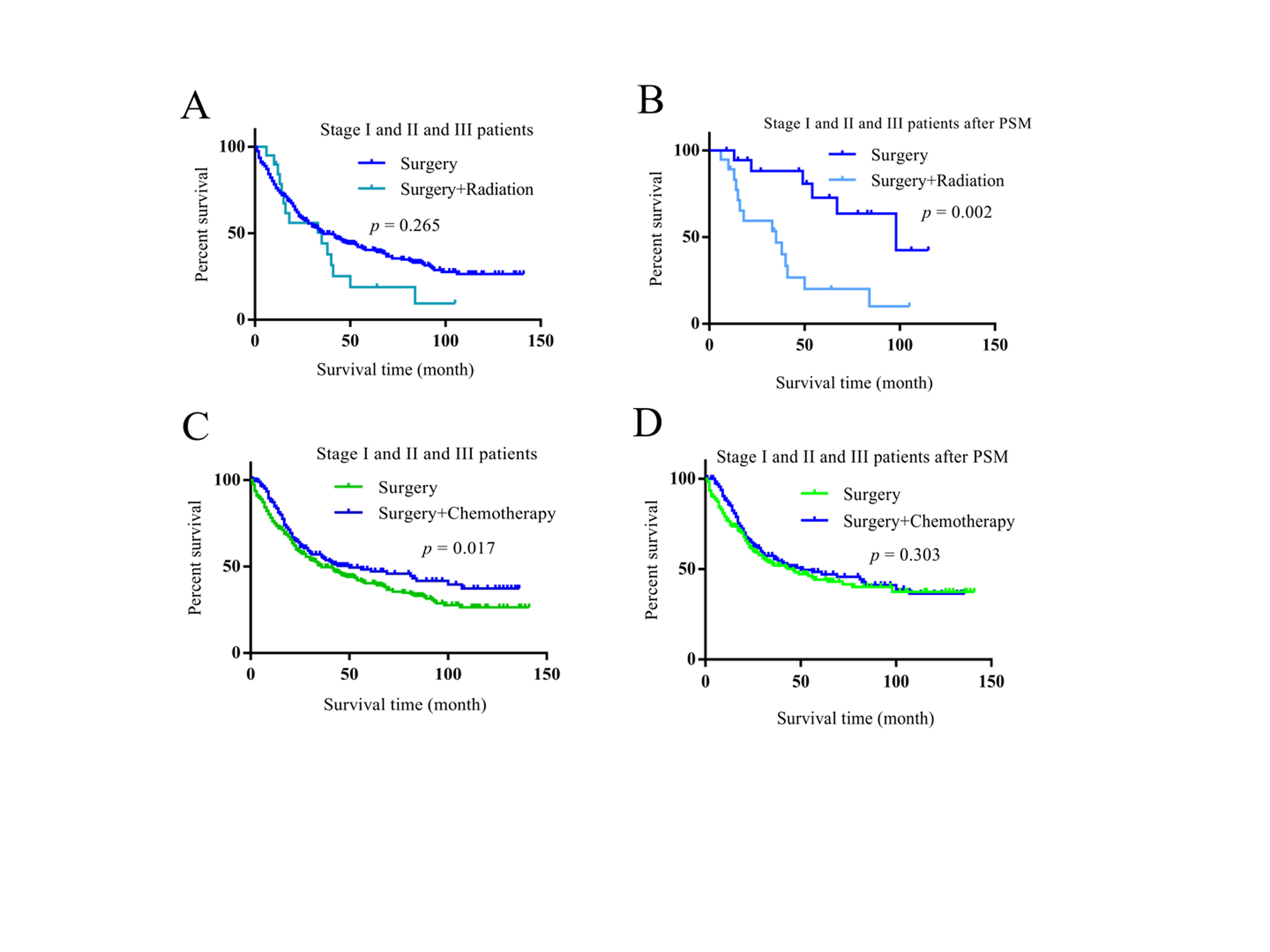

Supplement: Supplementary file 2 [file CAM4-8-2979-s002.tif]

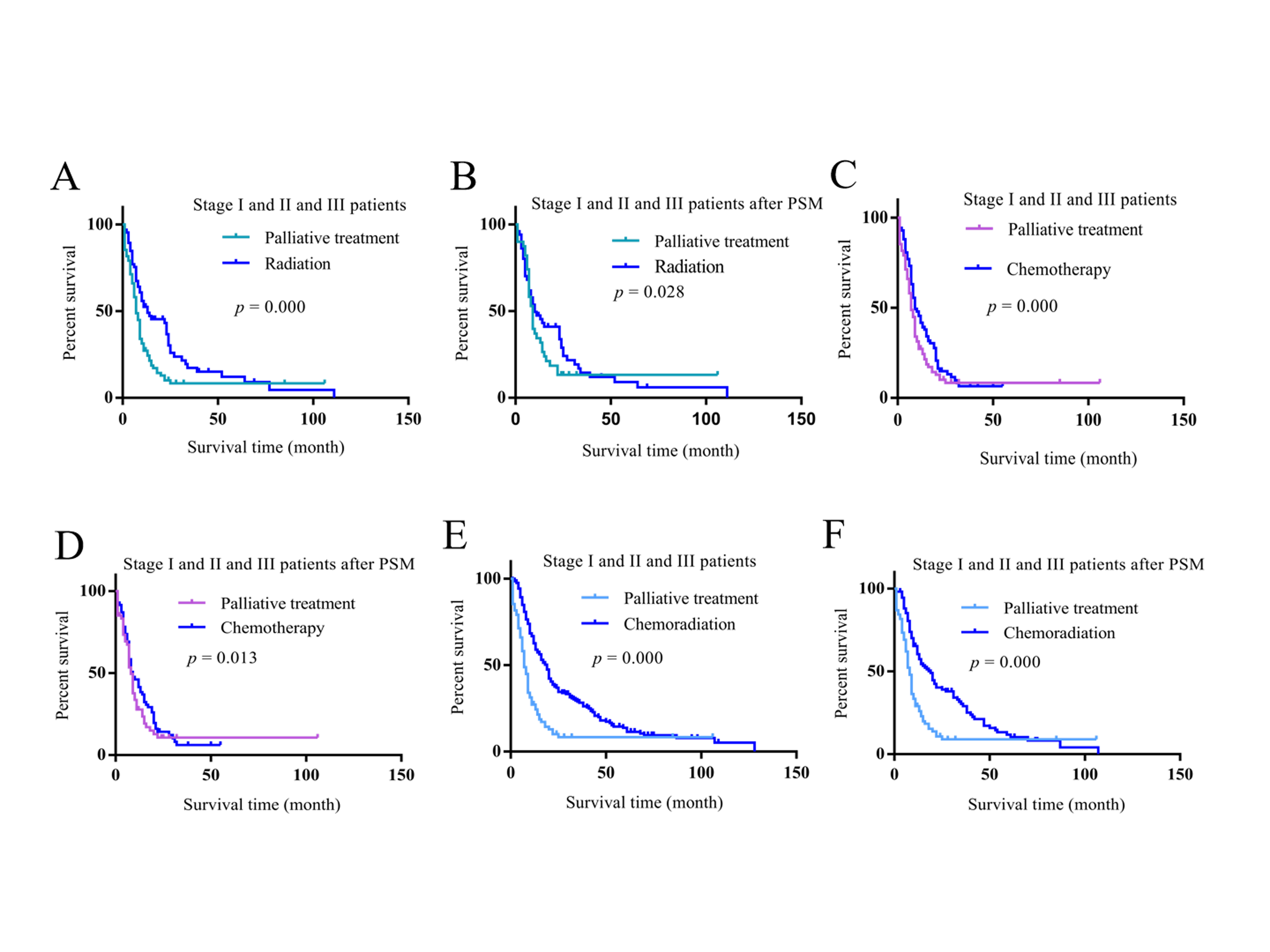

Supplement: Supplementary file 3 [file CAM4-8-2979-s003.tif]

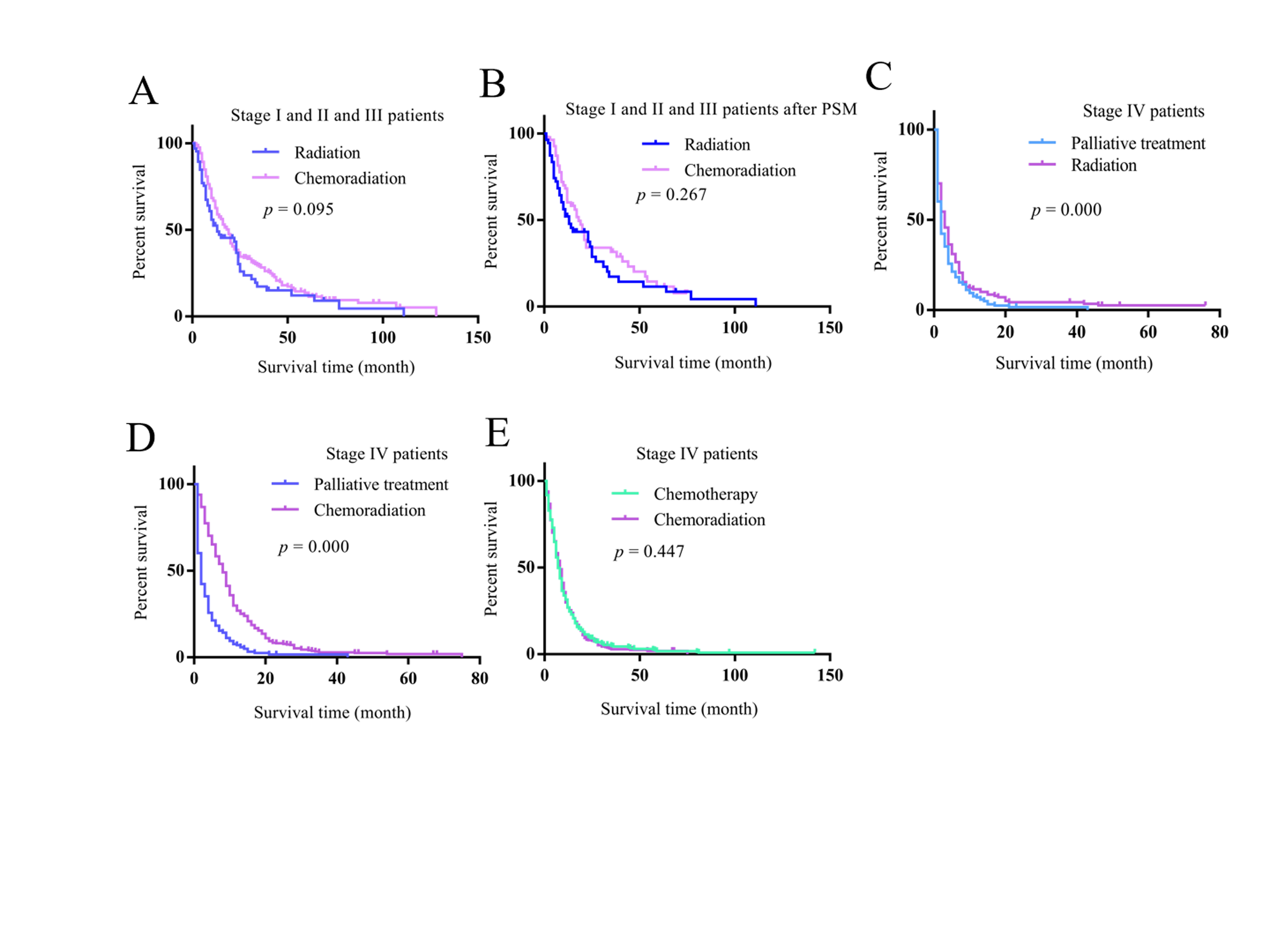

Supplement: Supplementary file 4 [file CAM4-8-2979-s004.tif]
