# Supplementary material for: The demographic and treatment options for patients with large cell neuroendocrine carcinoma of the lung
Source: Cancer Med. 2019 May 14;8(6):2979–93. doi: 10.1002/cam4.2188 (PMC6558599; doi:10.1002/cam4.2188)
Supplement: Supplementary file 5 [file CAM4-8-2979-s005.docx]

**Supplement table 1Characteristics among surgical and non-surgicalstage ⅢA LCNEC patients before and after propensity score matching**

| Characteristics |  | Before PSM analysis | | *p* |  | After PSM analysis | | *p* |
| --- | --- | --- | --- | --- | --- | --- | --- | --- |
|  |  | Non-S  (n=129) | S  (n=89) |  |  | Non-S  (n=56) | S  (n=56) |  |
| **Race** |  |  |  | 0.781 |  |  |  | 0.519 |
| White |  | 115 | 79 |  |  | 49 | 51 |  |
| Black |  | 12 | 6 |  |  | 6 | 4 |  |
| Others |  | 2 | 4 |  |  | 1 | 1 |  |
| **Age** |  |  |  | 0.828 |  |  |  | 0.684 |
| ≥60 |  | 91 | 94 |  |  | 38 | 40 |  |
| ＜60 |  | 38 | 35 |  |  | 18 | 16 |  |
| **Gender** |  |  |  | 0.374 |  |  |  | 0.707 |
| Male |  | 66 | 51 |  |  | 42 | 30 |  |
| Female |  | 63 | 38 |  |  | 24 | 26 |  |
| **Grade** |  |  |  | 0.000 |  |  |  | 0.919 |
| Ⅰ |  | 1 | 0 |  |  | 0 | 0 |  |
| Ⅱ |  | 0 | 3 |  |  | 0 | 0 |  |
| Ⅲ |  | 38 | 48 |  |  | 27 | 26 |  |
| Ⅳ |  | 11 | 13 |  |  | 8 | 9 |  |
| Unknown |  | 79 | 25 |  |  | 21 | 31 |  |
| **T** |  |  |  | 0.027 |  |  |  | 0.795 |
| Tx |  | 21 | 1 |  |  | 4 | 1 |  |
| T0 |  | 2 | 2 |  |  | 0 | 2 |  |
| T1 |  | 36 | 28 |  |  | 20 | 20 |  |
| T2 |  | 54 | 40 |  |  | 24 | 27 |  |
| T3 |  | 16 | 18 |  |  | 8 | 6 |  |
| T4 |  | 0 | 0 |  |  | 0 | 0 |  |
| **N** |  |  |  | 0.000 |  |  |  | 1.000 |
| Nx |  | 0 | 0 |  |  | 0 | 0 |  |
| N0 |  | 0 | 0 |  |  | 0 | 0 |  |
| N1 |  | 1 | 10 |  |  | 1 | 1 |  |
| N2 |  | 128 | 79 |  |  | 55 | 55 |  |
| N3 |  | 0 | 0 |  |  | 0 | 0 |  |
| **Radiation** |  |  |  | 0.000 |  |  |  | 1.000 |
| Yes |  | 90 | 38 |  |  | 30 | 30 |  |
| No |  | 39 | 51 |  |  | 26 | 26 |  |
| **Chemotherapy** |  |  |  | 0.414 |  |  |  | 0.387 |
| Yes |  | 95 | 61 |  |  | 44 | 40 |  |
| No |  | 34 | 28 |  |  | 12 | 16 |  |

**Supplement table 2Characteristics among surgical and non-surgicalstage ⅢB LCNEC patients before and after propensity score matching**

| Characteristics |  | Before PSM analysis | | *p* |  | After PSM analysis | | *p* |
| --- | --- | --- | --- | --- | --- | --- | --- | --- |
|  |  | Non-S  (n=256) | S  (n=51) |  |  | Non-S  (n=44) | S  (n=44) |  |
| **Race** |  |  |  | 0.492 |  |  |  | 0.873 |
| White |  | 201 | 42 |  |  | 37 | 36 |  |
| Black |  | 40 | 6 |  |  | 5 | 5 |  |
| Others |  | 15 | 3 |  |  | 2 | 3 |  |
| **Age** |  |  |  | 0.808 |  |  |  | 0.004 |
| ≥60 |  | 185 | 76 |  |  | 20 | 33 |  |
| ＜60 |  | 71 | 21 |  |  | 24 | 11 |  |
| **Gender** |  |  |  | 0.984 |  |  |  | 0.262 |
| Male |  | 156 | 31 |  |  | 32 | 27 |  |
| Female |  | 100 | 20 |  |  | 12 | 17 |  |
| **Grade** |  |  |  | 0.001 |  |  |  | 0.825 |
| Ⅰ |  | 1 | 1 |  |  | 0 | 1 |  |
| Ⅱ |  | 3 | 0 |  |  | 0 | 0 |  |
| Ⅲ |  | 80 | 29 |  |  | 24 | 24 |  |
| Ⅳ |  | 32 | 7 |  |  | 3 | 6 |  |
| Unknown |  | 140 | 14 |  |  | 17 | 13 |  |
| **T** |  |  |  | 0.276 |  |  |  | 0.559 |
| Tx |  | 16 | 0 |  |  | 2 | 0 |  |
| T0 |  | 1 | 0 |  |  | 0 | 0 |  |
| T1 |  | 6 | 0 |  |  | 0 | 0 |  |
| T2 |  | 23 | 2 |  |  | 4 | 2 |  |
| T3 |  | 6 | 0 |  |  | 1 | 0 |  |
| T4 |  | 204 | 49 |  |  | 37 | 42 |  |
| **N** |  |  |  | 0.000 |  |  |  | 0.830 |
| Nx |  | 9 | 0 |  |  | 2 | 0 |  |
| N0 |  | 41 | 29 |  |  | 16 | 22 |  |
| N1 |  | 9 | 5 |  |  | 2 | 5 |  |
| N2 |  | 113 | 13 |  |  | 12 | 13 |  |
| N3 |  | 84 | 4 |  |  | 12 | 4 |  |
| **Radiation** |  |  |  | 0.002 |  |  |  | 0.823 |
| Yes |  | 131 | 14 |  |  | 15 | 14 |  |
| No |  | 125 | 37 |  |  | 29 | 30 |  |
| **Chemotherapy** |  |  |  | 0.052 |  |  |  | 0.392 |
| Yes |  | 172 | 27 |  |  | 28 | 24 |  |
| No |  | 84 | 24 |  |  | 16 | 20 |  |

**Supplement table 3 Characteristics among surgical and non-surgicalstage Ⅳ LCNEC patients before and after propensity score matching**

| Characteristics |  | Before PSM analysis | | *p* |  | After PSM analysis | | *p* |
| --- | --- | --- | --- | --- | --- | --- | --- | --- |
|  |  | Non-S  (n=1278) | S  (n=87) |  |  | Non-S  (n=86) | S  (n=86) |  |
| **Race** |  |  |  | 0.616 |  |  |  | 0.822 |
| White |  | 1075 | 71 |  |  | 71 | 70 |  |
| Black |  | 147 | 11 |  |  | 10 | 11 |  |
| Others |  | 56 | 5 |  |  | 5 | 5 |  |
| **Age** |  |  |  | 0.564 |  |  |  | 0.000 |
| ≥60 |  | 904 | 59 |  |  | 13 | 58 |  |
| ＜60 |  | 374 | 28 |  |  | 73 | 28 |  |
| **Gender** |  |  |  | 0.686 |  |  |  | 0.879 |
| Male |  | 748 | 49 |  |  | 47 | 48 |  |
| Female |  | 530 | 38 |  |  | 39 | 38 |  |
| **Grade** |  |  |  | 0.000 |  |  |  | 0.473 |
| Ⅰ |  | 5 | 1 |  |  | 0 | 1 |  |
| Ⅱ |  | 6 | 2 |  |  | 0 | 1 |  |
| Ⅲ |  | 293 | 36 |  |  | 38 | 36 |  |
| Ⅳ |  | 95 | 17 |  |  | 6 | 17 |  |
| Unknown |  | 879 | 31 |  |  | 42 | 31 |  |
| **T** |  |  |  | 0.000 |  |  |  | 0.774 |
| Tx |  | 181 | 4 |  |  | 6 | 4 |  |
| T0 |  | 19 | 0 |  |  | 2 | 0 |  |
| T1 |  | 127 | 18 |  |  | 22 | 17 |  |
| T2 |  | 333 | 37 |  |  | 31 | 37 |  |
| T3 |  | 55 | 4 |  |  | 2 | 4 |  |
| T4 |  | 563 | 24 |  |  | 23 | 24 |  |
| **N** |  |  |  | 0.035 |  |  |  | 0.105 |
| Nx |  | 88 | 0 |  |  | 2 | 0 |  |
| N0 |  | 246 | 37 |  |  | 17 | 36 |  |
| N1 |  | 99 | 17 |  |  | 12 | 17 |  |
| N2 |  | 559 | 26 |  |  | 32 | 26 |  |
| N3 |  | 286 | 7 |  |  | 23 | 7 |  |
| **Radiation** |  |  |  | 0.254 |  |  |  | 0.029 |
| Yes |  | 595 | 46 |  |  | 59 | 45 |  |
| No |  | 683 | 41 |  |  | 27 | 41 |  |
| **Chemotherapy** |  |  |  | 0.747 |  |  |  | 0.025 |
| Yes |  | 757 | 50 |  |  | 63 | 49 |  |
| No |  | 521 | 37 |  |  | 23 | 37 |  |

**Supplement table 4 Characteristics among surgery alone and surgery combining with radiation in stage Ⅰ, Ⅱ and Ⅲ LCNEC patients before and after propensity score matching**

| Characteristics |  | Before PSM analysis | | *p* |  | After PSM analysis | | *p* |
| --- | --- | --- | --- | --- | --- | --- | --- | --- |
|  |  | S  (n=438) | S+R  (n=20) |  |  | S  (n=19) | S+R  (n=19) |  |
| **Race** |  |  |  | 0.912 |  |  |  | 0.266 |
| White |  | 368 | 16 |  |  | 18 | 15 |  |
| Black |  | 54 | 2 |  |  | 1 | 2 |  |
| Others |  | 16 | 2 |  |  | 0 | 2 |  |
| **Age** |  |  |  | 0.822 |  |  |  | 0.000 |
| ≥60 |  | 338 | 15 |  |  | 3 | 15 |  |
| ＜60 |  | 100 | 5 |  |  | 16 | 4 |  |
| **Gender** |  |  |  | 0.147 |  |  |  | 0.741 |
| Male |  | 226 | 7 |  |  | 6 | 7 |  |
| Female |  | 212 | 13 |  |  | 13 | 12 |  |
| **Grade** |  |  |  | 0.502 |  |  |  | 0.448 |
| Ⅰ |  | 3 | 0 |  |  | 2 | 0 |  |
| Ⅱ |  | 13 | 0 |  |  | 1 | 0 |  |
| Ⅲ |  | 236 | 9 |  |  | 7 | 8 |  |
| Ⅳ |  | 59 | 5 |  |  | 1 | 5 |  |
| Unknown |  | 127 | 6 |  |  | 8 | 6 |  |
| **TNM** |  |  |  | 0.145 |  |  |  | 0.149 |
| Stage Ⅰ |  | 354 | 13 |  |  | 17 | 13 |  |
| StageⅡ |  | 37 | 2 |  |  | 1 | 2 |  |
| Stage III |  | 47 | 5 |  |  | 1 | 4 |  |
| **T** |  |  |  | 0.038 |  |  |  | 0.015 |
| Tx |  | 0 | 0 |  |  | 0 | 0 |  |
| T0 |  | 2 | 0 |  |  | 0 | 0 |  |
| T1 |  | 232 | 7 |  |  | 16 | 7 |  |
| T2 |  | 172 | 7 |  |  | 2 | 7 |  |
| T3 |  | 11 | 5 |  |  | 0 | 4 |  |
| T4 |  | 21 | 1 |  |  | 1 | 1 |  |
| **N** |  |  |  | 0.113 |  |  |  | 0.350 |
| Nx |  | 0 | 0 |  |  | 0 | 0 |  |
| N0 |  | 376 | 15 |  |  | 17 | 15 |  |
| N1 |  | 32 | 4 |  |  | 1 | 3 |  |
| N2 |  | 28 | 1 |  |  | 1 | 1 |  |
| N3 |  | 2 | 0 |  |  | 0 | 0 |  |

**Supplement table 5 Characteristics among surgery alone and surgery combining with chemotherapy in stage Ⅰ, Ⅱ and Ⅲ LCNEC patients before and after propensity score matching**

| Characteristics |  | Before PSM analysis | | *p* |  | After PSM analysis | | *p* |
| --- | --- | --- | --- | --- | --- | --- | --- | --- |
|  |  | S  (n=438) | S+C  (n=195) |  |  | S  (n=171) | S+C  (n=171) |  |
| **Race** |  |  |  | 0.514 |  |  |  | 0.527 |
| White |  | 368 | 167 |  |  | 150 | 146 |  |
| Black |  | 54 | 20 |  |  | 14 | 17 |  |
| Others |  | 16 | 8 |  |  | 7 | 8 |  |
| **Age** |  |  |  | 0.000 |  |  |  | 0.049 |
| ≥60 |  | 338 | 115 |  |  | 89 | 107 |  |
| ＜60 |  | 100 | 80 |  |  | 82 | 64 |  |
| **Gender** |  |  |  | 0.216 |  |  |  | 0.663 |
| Male |  | 226 | 111 |  |  | 100 | 96 |  |
| Female |  | 212 | 84 |  |  | 71 | 75 |  |
| **Grade** |  |  |  | 0.178 |  |  |  | 0.460 |
| Ⅰ |  | 3 | 0 |  |  | 2 | 0 |  |
| Ⅱ |  | 13 | 4 |  |  | 6 | 4 |  |
| Ⅲ |  | 236 | 108 |  |  | 103 | 89 |  |
| Ⅳ |  | 59 | 45 |  |  | 22 | 42 |  |
| Unknown |  | 127 | 38 |  |  | 38 | 36 |  |
| **TNM** |  |  |  | 0.000 |  |  |  | 0.129 |
| Stage Ⅰ |  | 354 | 107 |  |  | 121 | 107 |  |
| StageⅡ |  | 37 | 47 |  |  | 25 | 31 |  |
| Stage III |  | 47 | 41 |  |  | 25 | 33 |  |
| **T** |  |  |  | 0.000 |  |  |  | 0.085 |
| Tx |  | 0 | 1 |  |  | 0 | 0 |  |
| T0 |  | 2 | 0 |  |  | 1 | 0 |  |
| T1 |  | 232 | 52 |  |  | 76 | 47 |  |
| T2 |  | 172 | 115 |  |  | 78 | 104 |  |
| T3 |  | 11 | 13 |  |  | 4 | 11 |  |
| T4 |  | 21 | 14 |  |  | 12 | 9 |  |
| **N** |  |  |  | 0.000 |  |  |  | 0.303 |
| Nx |  | 0 | 0 |  |  | 0 | 0 |  |
| N0 |  | 376 | 122 |  |  | 129 | 119 |  |
| N1 |  | 32 | 45 |  |  | 27 | 28 |  |
| N2 |  | 28 | 27 |  |  | 15 | 24 |  |
| N3 |  | 2 | 1 |  |  | 0 | 0 |  |

**Supplement table 6 Characteristics among palliative treatment and radiation in stage Ⅰ, Ⅱ and Ⅲ LCNEC patients before and after propensity score matching**

| Characteristics |  | Before PSM analysis | | *p* |  | After PSM analysis | | *p* |
| --- | --- | --- | --- | --- | --- | --- | --- | --- |
|  |  | P  (n=118) | R  (n=68) |  |  | P  (n=53) | R  (n=53) |  |
| **Race** |  |  |  | 0.175 |  |  |  | 1.000 |
| White |  | 103 | 54 |  |  | 43 | 43 |  |
| Black |  | 12 | 11 |  |  | 8 | 8 |  |
| Others |  | 3 | 3 |  |  | 2 | 2 |  |
| **Age** |  |  |  | 0.711 |  |  |  | 0.636 |
| ≥60 |  | 98 | 55 |  |  | 41 | 43 |  |
| ＜60 |  | 20 | 13 |  |  | 12 | 10 |  |
| **Gender** |  |  |  | 0.466 |  |  |  | 0.562 |
| Male |  | 69 | 36 |  |  | 31 | 28 |  |
| Female |  | 49 | 32 |  |  | 22 | 25 |  |
| **Grade** |  |  |  | 0.479 |  |  |  | 0.746 |
| Ⅰ |  | 0 | 1 |  |  | 0 | 1 |  |
| Ⅱ |  | 1 | 1 |  |  | 0 | 1 |  |
| Ⅲ |  | 30 | 21 |  |  | 12 | 13 |  |
| Ⅳ |  | 12 | 6 |  |  | 6 | 5 |  |
| Unknown |  | 75 | 39 |  |  | 35 | 33 |  |
| **TNM** |  |  |  | 0.000 |  |  |  | 0.710 |
| Stage Ⅰ |  | 25 | 32 |  |  | 18 | 18 |  |
| StageⅡ |  | 8 | 3 |  |  | 6 | 2 |  |
| Stage III |  | 85 | 33 |  |  | 29 | 33 |  |
| **T** |  |  |  | 0.008 |  |  |  | 0.186 |
| Tx |  | 12 | 6 |  |  | 7 | 6 |  |
| T0 |  | 0 | 1 |  |  | 0 | 1 |  |
| T1 |  | 21 | 21 |  |  | 18 | 13 |  |
| T2 |  | 25 | 21 |  |  | 16 | 15 |  |
| T3 |  | 6 | 3 |  |  | 4 | 2 |  |
| T4 |  | 54 | 16 |  |  | 8 | 16 |  |
| **N** |  |  |  | 0.018 |  |  |  | 0.699 |
| Nx |  | 4 | 1 |  |  | 1 | 1 |  |
| N0 |  | 42 | 36 |  |  | 22 | 21 |  |
| N1 |  | 7 | 2 |  |  | 4 | 2 |  |
| N2 |  | 53 | 21 |  |  | 25 | 21 |  |
| N3 |  | 12 | 8 |  |  | 1 | 8 |  |

**Supplement table 7 Characteristics among palliative treatment and chemotherapy in stage Ⅰ, Ⅱ and Ⅲ LCNEC patients before and after propensity score matching**

| Characteristics |  | Before PSM analysis | | *p* |  | After PSM analysis | | *p* |
| --- | --- | --- | --- | --- | --- | --- | --- | --- |
|  |  | P  (n=118) | C  (n=91) |  |  | P  (n=77) | R  (n=77) |  |
| **Race** |  |  |  | 0.067 |  |  |  | 0.553 |
| White |  | 103 | 70 |  |  | 63 | 65 |  |
| Black |  | 12 | 16 |  |  | 11 | 8 |  |
| Others |  | 3 | 5 |  |  | 3 | 4 |  |
| **Age** |  |  |  | 0.067 |  |  |  | 1.000 |
| ≥60 |  | 98 | 66 |  |  | 58 | 58 |  |
| ＜60 |  | 20 | 25 |  |  | 19 | 19 |  |
| **Gender** |  |  |  | 0.656 |  |  |  | 0.622 |
| Male |  | 69 | 56 |  |  | 49 | 46 |  |
| Female |  | 49 | 35 |  |  | 28 | 31 |  |
| **Grade** |  |  |  | 0.038 |  |  |  | 0.860 |
| Ⅰ |  | 0 | 0 |  |  | 0 | 0 |  |
| Ⅱ |  | 1 | 1 |  |  | 1 | 0 |  |
| Ⅲ |  | 30 | 32 |  |  | 24 | 24 |  |
| Ⅳ |  | 12 | 15 |  |  | 9 | 14 |  |
| Unknown |  | 75 | 43 |  |  | 43 | 39 |  |
| **TNM** |  |  |  | 0.007 |  |  |  | 0.719 |
| Stage Ⅰ |  | 25 | 6 |  |  | 9 | 6 |  |
| StageⅡ |  | 8 | 6 |  |  | 7 | 4 |  |
| Stage III |  | 85 | 79 |  |  | 61 | 67 |  |
| **T** |  |  |  | 0.272 |  |  |  | 0.313 |
| Tx |  | 12 | 7 |  |  | 10 | 6 |  |
| T0 |  | 0 | 0 |  |  | 0 | 0 |  |
| T1 |  | 21 | 6 |  |  | 17 | 5 |  |
| T2 |  | 25 | 23 |  |  | 11 | 18 |  |
| T3 |  | 6 | 8 |  |  | 5 | 7 |  |
| T4 |  | 54 | 47 |  |  | 34 | 41 |  |
| **N** |  |  |  | 0.070 |  |  |  | 0.901 |
| Nx |  | 4 | 2 |  |  | 2 | 2 |  |
| N0 |  | 42 | 17 |  |  | 20 | 14 |  |
| N1 |  | 7 | 4 |  |  | 7 | 3 |  |
| N2 |  | 53 | 43 |  |  | 41 | 39 |  |
| N3 |  | 12 | 25 |  |  | 7 | 19 |  |

**Supplement table 8 Characteristics among palliative treatment and chemoradiation in stage Ⅰ, Ⅱ and Ⅲ LCNEC patients before and after propensity score matching**

| Characteristics |  | Before PSM analysis | | *p* |  | After PSM analysis | | *p* |
| --- | --- | --- | --- | --- | --- | --- | --- | --- |
|  |  | P  (n=118) | C+R  (n=215) |  |  | P  (n=112) | C+R  (n=112) |  |
| **Race** |  |  |  | 0.148 |  |  |  | 0.478 |
| White |  | 103 | 174 |  |  | 97 | 92 |  |
| Black |  | 12 | 33 |  |  | 12 | 14 |  |
| Others |  | 3 | 8 |  |  | 3 | 6 |  |
| **Age** |  |  |  | 0.005 |  |  |  | 0.723 |
| ≥60 |  | 98 | 148 |  |  | 92 | 94 |  |
| ＜60 |  | 20 | 67 |  |  | 20 | 18 |  |
| **Gender** |  |  |  | 0.429 |  |  |  | 0.139 |
| Male |  | 69 | 116 |  |  | 69 | 58 |  |
| Female |  | 49 | 99 |  |  | 43 | 54 |  |
| **Grade** |  |  |  | 0.302 |  |  |  | 0.825 |
| Ⅰ |  | 0 | 1 |  |  | 0 | 1 |  |
| Ⅱ |  | 1 | 1 |  |  | 1 | 1 |  |
| Ⅲ |  | 30 | 68 |  |  | 30 | 29 |  |
| Ⅳ |  | 12 | 22 |  |  | 12 | 12 |  |
| Unknown |  | 75 | 123 |  |  | 69 | 69 |  |
| **TNM** |  |  |  | 0.025 |  |  |  | 0.870 |
| Stage Ⅰ |  | 25 | 21 |  |  | 23 | 18 |  |
| StageⅡ |  | 8 | 6 |  |  | 8 | 0 |  |
| Stage III |  | 85 | 188 |  |  | 81 | 94 |  |
| **T** |  |  |  | 0.241 |  |  |  | 0.919 |
| Tx |  | 12 | 12 |  |  | 12 | 8 |  |
| T0 |  | 0 | 2 |  |  | 0 | 2 |  |
| T1 |  | 21 | 38 |  |  | 21 | 11 |  |
| T2 |  | 25 | 59 |  |  | 23 | 38 |  |
| T3 |  | 6 | 17 |  |  | 6 | 4 |  |
| T4 |  | 54 | 87 |  |  | 50 | 49 |  |
| **N** |  |  |  | 0.004 |  |  |  | 0.884 |
| Nx |  | 4 | 2 |  |  | 3 | 1 |  |
| N0 |  | 42 | 42 |  |  | 37 | 32 |  |
| N1 |  | 7 | 8 |  |  | 7 | 0 |  |
| N2 |  | 53 | 124 |  |  | 53 | 58 |  |
| N3 |  | 12 | 39 |  |  | 12 | 21 |  |

**Supplement table 9 Characteristics among radiation and chemoradiation in stage Ⅰ, Ⅱ and Ⅲ LCNEC patients before and after propensity score matching**

| Characteristics |  | Before PSM analysis | | *p* |  | After PSM analysis | | *p* |
| --- | --- | --- | --- | --- | --- | --- | --- | --- |
|  |  | R  (n=68) | C+R  (n=215) |  |  | R  (n=57) | C+R  (n=57) |  |
| **Race** |  |  |  | 0.819 |  |  |  | 0.711 |
| White |  | 54 | 174 |  |  | 46 | 46 |  |
| Black |  | 11 | 33 |  |  | 8 | 11 |  |
| Others |  | 3 | 8 |  |  | 3 | 0 |  |
| **Age** |  |  |  | 0.055 |  |  |  | 0.639 |
| ≥60 |  | 55 | 148 |  |  | 47 | 94 |  |
| ＜60 |  | 13 | 67 |  |  | 10 | 18 |  |
| **Gender** |  |  |  | 0.884 |  |  |  | 0.189 |
| Male |  | 36 | 116 |  |  | 29 | 36 |  |
| Female |  | 32 | 99 |  |  | 28 | 21 |  |
| **Grade** |  |  |  | 0.946 |  |  |  | 0.521 |
| Ⅰ |  | 1 | 1 |  |  | 1 | 0 |  |
| Ⅱ |  | 1 | 1 |  |  | 0 | 0 |  |
| Ⅲ |  | 21 | 68 |  |  | 13 | 15 |  |
| Ⅳ |  | 6 | 22 |  |  | 6 | 7 |  |
| Unknown |  | 39 | 123 |  |  | 37 | 35 |  |
| **TNM** |  |  |  | 0.000 |  |  |  | 0.854 |
| Stage Ⅰ |  | 32 | 21 |  |  | 21 | 21 |  |
| StageⅡ |  | 3 | 6 |  |  | 3 | 1 |  |
| Stage III |  | 33 | 188 |  |  | 33 | 35 |  |
| **T** |  |  |  | 0.038 |  |  |  | 0.734 |
| Tx |  | 6 | 12 |  |  | 6 | 1 |  |
| T0 |  | 1 | 2 |  |  | 1 | 0 |  |
| T1 |  | 21 | 38 |  |  | 18 | 11 |  |
| T2 |  | 21 | 59 |  |  | 13 | 24 |  |
| T3 |  | 3 | 17 |  |  | 3 | 0 |  |
| T4 |  | 16 | 87 |  |  | 16 | 21 |  |
| **N** |  |  |  | 0.000 |  |  |  | 0.750 |
| Nx |  | 1 | 2 |  |  | 1 | 0 |  |
| N0 |  | 36 | 42 |  |  | 25 | 28 |  |
| N1 |  | 2 | 8 |  |  | 2 | 1 |  |
| N2 |  | 21 | 124 |  |  | 21 | 23 |  |
| N3 |  | 8 | 39 |  |  | 8 | 5 |  |

**Supplement table 10 Characteristics among palliative treatment and radiation in Ⅳ LCNEC patients before propensity score matching**

| Characteristics |  | Before PSM analysis | | *p* |  |
| --- | --- | --- | --- | --- | --- |
|  |  | P  (n=337) | R  (n=184) |  |  |
| **Race** |  |  |  | 0.120 |  |
| White |  | 294 | 151 |  |  |
| Black |  | 31 | 24 |  |  |
| Others |  | 12 | 9 |  |  |
| **Age** |  |  |  | 0.248 |  |
| ≥60 |  | 271 | 140 |  |  |
| ＜60 |  | 66 | 44 |  |  |
| **Gender** |  |  |  | 0.156 |  |
| Male |  | 203 | 99 |  |  |
| Female |  | 134 | 85 |  |  |
| **Grade** |  |  |  | 0.332 |  |
| Ⅰ |  | 0 | 0 |  |  |
| Ⅱ |  | 1 | 1 |  |  |
| Ⅲ |  | 67 | 42 |  |  |
| Ⅳ |  | 23 | 14 |  |  |
| Unknown |  | 246 | 127 |  |  |
| **T** |  |  |  | 0.372 |  |
| Tx |  | 54 | 23 |  |  |
| T0 |  | 4 | 3 |  |  |
| T1 |  | 34 | 19 |  |  |
| T2 |  | 80 | 49 |  |  |
| T3 |  | 13 | 10 |  |  |
| T4 |  | 152 | 80 |  |  |
| **N** |  |  |  | 0.160 |  |
| Nx |  | 35 | 13 |  |  |
| N0 |  | 58 | 47 |  |  |
| N1 |  | 31 | 16 |  |  |
| N2 |  | 149 | 77 |  |  |
| N3 |  | 64 | 31 |  |  |

**Supplement table 11 Characteristics among palliative treatment and chemoradiation in Ⅳ LCNEC patients before and after propensity score matching**

| Characteristics |  | Before PSM analysis | | *p* |  | After PSM analysis | | *p* |
| --- | --- | --- | --- | --- | --- | --- | --- | --- |
|  |  | P  (n=337) | C+R  (n=411) |  |  | P  (n=297) | C+R  (n=297) |  |
| **Race** |  |  |  | 0.032 |  |  |  | 0.947 |
| White |  | 294 | 335 |  |  | 255 | 253 |  |
| Black |  | 31 | 57 |  |  | 30 | 29 |  |
| Others |  | 12 | 19 |  |  | 12 | 15 |  |
| **Age** |  |  |  | 0.000 |  |  |  | 0.768 |
| ≥60 |  | 271 | 239 |  |  | 232 | 229 |  |
| ＜60 |  | 66 | 172 |  |  | 65 | 68 |  |
| **Gender** |  |  |  | 0.399 |  |  |  | 0.804 |
| Male |  | 203 | 235 |  |  | 171 | 168 |  |
| Female |  | 134 | 176 |  |  | 126 | 129 |  |
| **Grade** |  |  |  | 0.213 |  |  |  | 0.923 |
| Ⅰ |  | 0 | 1 |  |  | 0 | 1 |  |
| Ⅱ |  | 1 | 2 |  |  | 1 | 1 |  |
| Ⅲ |  | 67 | 97 |  |  | 65 | 65 |  |
| Ⅳ |  | 23 | 28 |  |  | 21 | 20 |  |
| Unknown |  | 247 | 283 |  |  | 210 | 210 |  |
| **T** |  |  |  | 0.171 |  |  |  | 0.950 |
| Tx |  | 54 | 44 |  |  | 42 | 34 |  |
| T0 |  | 4 | 9 |  |  | 3 | 6 |  |
| T1 |  | 34 | 45 |  |  | 33 | 31 |  |
| T2 |  | 80 | 111 |  |  | 76 | 77 |  |
| T3 |  | 13 | 19 |  |  | 13 | 13 |  |
| T4 |  | 152 | 183 |  |  | 130 | 136 |  |
| **N** |  |  |  | 0.369 |  |  |  | 0.871 |
| Nx |  | 35 | 19 |  |  | 27 | 13 |  |
| N0 |  | 58 | 87 |  |  | 54 | 61 |  |
| N1 |  | 31 | 28 |  |  | 25 | 20 |  |
| N2 |  | 149 | 176 |  |  | 134 | 139 |  |
| N3 |  | 64 | 101 |  |  | 57 | 64 |  |

**Supplement table 12 Characteristics among radiation and chemoradiation in Ⅳ LCNEC patients before and after propensity score matching**

| Characteristics |  | Before PSM analysis | | *p* |  | After PSM analysis | | *p* |
| --- | --- | --- | --- | --- | --- | --- | --- | --- |
|  |  | R  (n=184) | C+R  (n=411) |  |  | R  (n=184) | C+R  (n=184) |  |
| **Race** |  |  |  | 0.824 |  |  |  | 0.389 |
| White |  | 151 | 335 |  |  | 151 | 158 |  |
| Black |  | 24 | 57 |  |  | 24 | 20 |  |
| Others |  | 9 | 19 |  |  | 9 | 6 |  |
| **Age** |  |  |  | 0.000 |  |  |  | 1.000 |
| ≥60 |  | 140 | 239 |  |  | 140 | 140 |  |
| ＜60 |  | 44 | 172 |  |  | 44 | 44 |  |
| **Gender** |  |  |  | 0.444 |  |  |  | 0.096 |
| Male |  | 99 | 235 |  |  | 99 | 83 |  |
| Female |  | 85 | 176 |  |  | 85 | 101 |  |
| **Grade** |  |  |  | 0.965 |  |  |  | 0.441 |
| Ⅰ |  | 0 | 1 |  |  | 0 | 1 |  |
| Ⅱ |  | 1 | 2 |  |  | 1 | 0 |  |
| Ⅲ |  | 42 | 97 |  |  | 42 | 51 |  |
| Ⅳ |  | 14 | 28 |  |  | 14 | 13 |  |
| Unknown |  | 127 | 283 |  |  | 127 | 119 |  |
| **T** |  |  |  | 0.831 |  |  |  | 0.129 |
| Tx |  | 23 | 44 |  |  | 23 | 19 |  |
| T0 |  | 3 | 9 |  |  | 3 | 2 |  |
| T1 |  | 19 | 45 |  |  | 19 | 31 |  |
| T2 |  | 49 | 111 |  |  | 49 | 49 |  |
| T3 |  | 10 | 19 |  |  | 10 | 7 |  |
| T4 |  | 80 | 183 |  |  | 80 | 76 |  |
| **N** |  |  |  | 0.465 |  |  |  | 0.603 |
| Nx |  | 13 | 19 |  |  | 13 | 8 |  |
| N0 |  | 47 | 87 |  |  | 47 | 39 |  |
| N1 |  | 16 | 28 |  |  | 16 | 7 |  |
| N2 |  | 77 | 176 |  |  | 77 | 87 |  |
| N3 |  | 31 | 101 |  |  | 31 | 43 |  |

**Supplement table 13 Characteristics among chemotherapy and chemoradiation in stage Ⅳ LCNEC patients before and after propensity score matching**

| Characteristics |  | Before PSM analysis | | *p* |  | After PSM analysis | | *p* |
| --- | --- | --- | --- | --- | --- | --- | --- | --- |
|  |  | C  (n=346) | C+R  (n=411) |  |  | C  (n=313) | C+R  (n=313) |  |
| **Race** |  |  |  | 0.125 |  |  |  | 0.951 |
| White |  | 292 | 335 |  |  | 264 | 264 |  |
| Black |  | 35 | 57 |  |  | 34 | 35 |  |
| Others |  | 16 | 19 |  |  | 15 | 14 |  |
| **Age** |  |  |  | 0.000 |  |  |  | 1.000 |
| ≥60 |  | 254 | 239 |  |  | 221 | 221 |  |
| ＜60 |  | 92 | 172 |  |  | 92 | 92 |  |
| **Gender** |  |  |  | 0.290 |  |  |  | 0.935 |
| Male |  | 211 | 235 |  |  | 185 | 186 |  |
| Female |  | 135 | 176 |  |  | 128 | 127 |  |
| **Grade** |  |  |  | 0.503 |  |  |  | 0.893 |
| Ⅰ |  | 4 | 1 |  |  | 4 | 0 |  |
| Ⅱ |  | 2 | 2 |  |  | 2 | 2 |  |
| Ⅲ |  | 87 | 97 |  |  | 77 | 79 |  |
| Ⅳ |  | 30 | 28 |  |  | 27 | 22 |  |
| Unknown |  | 223 | 283 |  |  | 203 | 210 |  |
| **T** |  |  |  | 0.179 |  |  |  | 0.668 |
| Tx |  | 60 | 44 |  |  | 55 | 33 |  |
| T0 |  | 3 | 9 |  |  | 3 | 7 |  |
| T1 |  | 29 | 45 |  |  | 29 | 29 |  |
| T2 |  | 93 | 111 |  |  | 87 | 86 |  |
| T3 |  | 13 | 19 |  |  | 13 | 15 |  |
| T4 |  | 148 | 183 |  |  | 126 | 143 |  |
| **N** |  |  |  | 0.162 |  |  |  | 0.583 |
| Nx |  | 21 | 19 |  |  | 21 | 12 |  |
| N0 |  | 54 | 87 |  |  | 53 | 66 |  |
| N1 |  | 24 | 28 |  |  | 24 | 24 |  |
| N2 |  | 157 | 176 |  |  | 135 | 135 |  |
| N3 |  | 90 | 101 |  |  | 80 | 76 |  |
